# Prevalence of low muscle mass and associated factors in community-dwelling older adults in Singapore

**DOI:** 10.1038/s41598-021-02274-3

**Published:** 2021-11-29

**Authors:** Siew Ling Tey, Dieu Thi Thu Huynh, Yatin Berde, Geraldine Baggs, Choon How How, Yen Ling Low, Magdalin Cheong, Wai Leng Chow, Ngiap Chuan Tan, Samuel Teong Huang Chew

**Affiliations:** 1 Abbott Nutrition Research and Development, Asia-Pacific Center, Singapore, 138668 Singapore; 2 Statistical Services, Cognizant Technologies Solution Pvt. Ltd., Mumbai, India; 3 Abbott Nutrition Research and Development, Columbus, OH 43219 USA; 4 Care and Health Integration, Changi General Hospital, Singapore, 529889 Singapore; 5 SingHealth-Duke NUS Family Medicine Academic Clinical Program, Duke-NUS Medical School, Singapore, 169857 Singapore; 6 Department of Dietetic & Food Services, Changi General Hospital, Singapore, 529889 Singapore; 7 Health Services Research, Changi General Hospital, Singapore, 529889 Singapore; 8 SingHealth Polyclinics, Singapore, 150167 Singapore; 9 Department of Geriatric Medicine, Changi General Hospital, Singapore, 529889 Singapore; 10 Yong Loo Lin School of Medicine, National University of Singapore, Singapore, 117597 Singapore

**Keywords:** Geriatrics, Nutrition

## Abstract

The population is rapidly aging worldwide, and there is an age-related decline in muscle mass. Therefore, it is important to examine the prevalence and associated factors of low appendicular skeletal muscle mass index (ASMI) in older adults. The objectives of this cross-sectional study were (i) to determine the prevalence of low ASMI (ASM/height^2^) and (ii) to identify factors associated with low ASMI. This study included 1211 community-dwelling adults aged ≥ 65 years. Low ASMI was defined as < 7.0 kg/m^2^ in males and < 5.7 kg/m^2^ in females (bioelectrical impedance analysis). Gender-specific cut-off values of calf circumference for low ASMI were determined. The prevalence of low ASMI in the overall cohort was 59.9%, i.e., 57.0% among males and 61.8% among females, with no significant difference between genders (*P* = 0.1068). The prevalence of low ASMI was 81.3% in individuals at risk of malnutrition compared to 20.6% in their counterparts with normal nutritional status (*P* < 0.0001). Participants with low ASMI were older, had lower physical activity scores, and greater likelihood of hospitalization in prior 6 months compared with normal ASMI (all *P* < 0.0001). Low ASMI was associated with risk of malnutrition (odds ratio: 3.58 for medium risk, odds ratio: 12.50 for high risk), older age, smoking, drinking, smaller calf circumference, and lower bone mass (all *P* ≤ 0.0328). Cut-off values of calf circumference for low ASMI for males was 33.4 cm and for females was 32.2 cm. In conclusion, we found that low ASMI was highly prevalent among community-dwelling older adults at risk of malnutrition. Other significant factors associated with low ASMI were age, smoking, drinking, calf circumference, and bone mass. Screening community-dwelling older adults for risk of malnutrition can prevent or delay onset of low ASMI.

## Introduction

Aging is associated with physiological decline in skeletal muscle mass, which can escalate the tolls on overall health and function, and may ultimately increase risk of death^1–3^. Across Asia, low muscle mass is reported to occur commonly in community-dwelling older adults^4–8^, ranging from 20 to 63%. It is also known that women have lower muscle mass than men^9^. Since age-related muscle decline occurs in both women and men, women remain to have lower muscle mass than men in older age^10–13^. Thus, gender-specific measurements and cut-offs are important for more accurate identifications of low muscle mass, especially for surrogate marker of muscle mass such as calf circumference.

**Figure 1:**
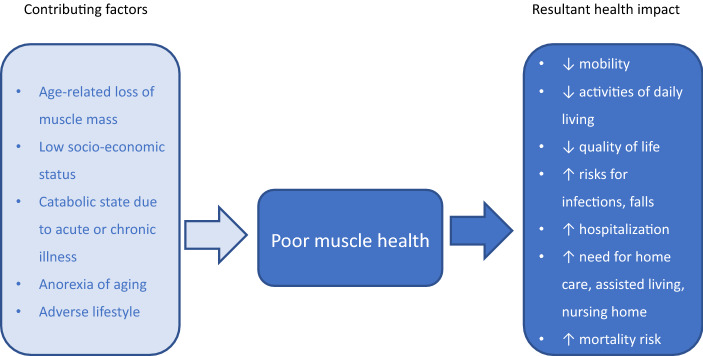
Factors contributing to poor muscle health and its impact in older adults.

Factors predisposing older adults to poor muscle health are low socio-economic status, underlying chronic diseases, poor nutritional intake, and adverse lifestyle (Fig. 1). Socio-economic deprivation includes low income and low education level^14,15^. Chronic diseases which can increase the risk of muscle decline include diabetes and chronic obstructive pulmonary disease^16,17^. In addition, acute illness or injury can initiate a cascade ultimately leading to poor muscle health^18^. In older adults, anorexia of aging is associated with low muscle mass and strength^19^. Lifestyle-related risks such as physical inactivity^20^, smoking^21^, and drinking^14^ are also associated with poor muscle health.

Older adults with evidence of low muscle mass are at high risk of adverse health outcomes such as reduced mobility, impaired ability to perform activities of daily living and lower quality of life, fall-related injuries, infections, hospitalization and need for long-term care^3,4^ (Fig. 1). A vicious cycle may then develop resulting in more loss of muscle mass, further disability, poorer health, ultimately leading to increased mortality risk^2,3^. However, there is recognized heterogeneity in the literature findings on the impact of muscle mass on functional ability in older adults^22,23^.

In a recent narrative review, low muscle mass or sarcopenia was consistently predictive of higher healthcare expenditure in community, perioperative, and general hospital settings^24^. In a study of older people in Taiwan, men and women with a lower skeletal muscle mass index (SMMI) had more hospitalizations, longer lengths of stay, and higher healthcare costs^25^. The annual difference in hospitalization costs between individuals with highest and lowest risk for low SMMI was equivalent to about United States dollars (USD) $1200 per patient year^25^. A United States study estimated direct healthcare costs for people with sarcopenia at 1.5% of the total annual healthcare expenditure, i.e., costs equivalent to USD $26.3 billion in 2020^26^. A more recent estimate of US costs for hospitalization in individuals with sarcopenia was USD $40.4 billion^27^.

Singapore’s population is growing older, similarly, but even faster than populations worldwide^28^. The global population of people ≥ 65 years has been projected to double from 703 million in 2019 to 1.5 billion by 2050^28^. Of this total, approximately 1.0 billion older adults will be living in Asia. In Singapore, the percentage of older adults is expected to double over the next decade from 12.4% in 2019 to 22.5% in 2030^28^. Thus, the objectives of this cross-sectional study were: (1) to determine the prevalence of low appendicular skeletal muscle mass index (ASMI; ASM/height^2^), and (2) to identify factors associated with low ASMI. The findings from this study may help to identify community-dwelling older adults at risk of low ASMI, in order to prevent and address the adverse consequences.

## Methods

### Study design and participants

Strengthening Health In ELDerly through nutrition (SHIELD) was a cross-sectional study of community-dwelling older adults in Singapore. Participants were recruited between August 2017 and March 2019. All procedures involving human subjects were approved by the Centralized Institutional Review Board in Singapore (reference number: 2017/2271). The present study was conducted according to the ethical standards laid down in the Declaration of Helsinki. Written informed consent was obtained from all participants. The trial was registered at clinicaltrials.gov as NCT03240952. The Nutritional Health for the Elderly Reference Centre (NHERC) is the name of the overarching project. One component of NHERC is the clinical trial, which is known as the SHIELD study.

A total of 1211 individuals consisting of 400 with normal nutritional status and 811 at risk of malnutrition took part in this study. The data for this study was derived from baseline measurements of two unique cohorts of the SHIELD study^29,30^. Participants were recruited from the public, community centers, polyclinics, and hospitals through public talks, flyers, posters, and referrals from healthcare professionals.

### Assessments

All study participants were asked to attend one visit at baseline, where the following data were collected.

Socio-demographic data such as age, gender, ethnicity, marital status, education, number of prescribed drugs, smoker status, and alcohol consumption were collected during the visit. Charlson Comorbidity Index was used to determine the comorbidity level^31,32^, and Malnutrition Universal Screening Test (MUST) was used to determine the risk of malnutrition^33^.

Physical Activity Scale for the Elderly (PASE)^34,35^ was used to determine physical activity level and Modified Barthel Index (MBI)^36^ was used to measure functional independence for ten activities of daily living.

For anthropometry and body composition measurements, standing height was measured without shoes by using a stadiometer to the nearest millimeter (Avamech B1000), and body weight and composition were measured to the nearest 0.1 kg using a bioelectrical impedance analysis (BIA) machine (Tanita MC-780). BIA was used to estimate muscle mass, fat mass, and bone mass. Mid upper arm circumference was measured at mid-point of the acromion and olecranon, and calf circumference was measured at the largest part of the calf. Both were measured to the nearest 0.1 cm.

Serum 25-hydroxyvitamin D levels were measured using immunochemistry analyzer COBAS e801 and vitamin D cut-off values were based on the definition described by Holick^37^.

Healthcare utilization data on hospitalization and length of stay was collected using medical records and questionnaires if the former is unavailable.

### Statistical analysis

Baseline characteristics of the study participants were reported as means and standard deviation (SD) for continuous variables, and numbers and percentages for categorical variables. For continuous variables, normality of the data was assessed using the Shapiro-Wilks test (*P* < 0.001) and graphical methods. Two-sample t test, Wilcoxon rank sum test, fisher exact test, and chi-square test were used to compare the characteristics between genders, nutritional status, and ASMI status. Low ASMI was determined based on BIA cut-offs recommended by the Asian Working Group for Sarcopenia (AWGS)^1^, i.e., male < 7.0 kg/m^2^ and female < 5.7 kg/m^2^.

Multiple linear regression model was used to examine the associations between ASMI and all potential variables based on the literature. The adjusted R-square values measuring goodness of model fit were ≥ 0.6 for all the three models (overall cohort, males, and females). Multicollinearity between the predictors were tested using variance inflation factor and tolerance. The highest variance inflation factor (VIF) was bone mass with 3.6 VIF and the lowest tolerance was 0.3, which confirmed that there was no multicollinearity. Multiple logistic regression model was used to examine the associations between low ASMI and potential factors. Odds ratio and 95% confidence interval (CI) were estimated from logistic regression models.

Logistic regression and Receiver Operating Characteristics (ROC) method were used to determine the cut-off values for calf circumference associated with low ASMI in males and females. Kappa statistics was used to test the agreement between the cut-off values for low ASMI in the present study and the cut-off values for screening for sarcopenia recommended by the AWGS^1^, i.e., male < 34 cm and female < 33 cm.

SAS version 9.4 (SAS Institute, Cary, NC, USA) was used for all statistical analyses. *P* < 0.0500 was considered statistically significant.

### Institutional review board statement

The study was conducted according to the guidelines of the Declaration of Helsinki, and approved by the Centralized Institutional Review Board in Singapore of SingHealth (reference number: 2017/2271 and date of approval: 23 May 2017).

### Informed consent statement

Informed consent was obtained from all subjects involved in the study.

## Results

### Participants’ characteristics

Table 1 shows the characteristics of the overall cohort, males, and females. A total of 1211 older adults participated in this study. Mean (SD) age of participants was 73.20 (6.82) years and body mass index (BMI) was 20.43 (3.69) kg/m^2^. Most participants were Chinese (85.5%) and over 94% had a Charlson Comorbidity score of 0. More than half of the participants (57.0%) had either vitamin D deficiency or insufficiency. The prevalence of low ASMI in the overall cohort was nearly 60%, with no significant difference between genders (57.0% males vs. 61.8% females, *P* = 0.1068). Supplementary Table 1 shows the characteristics of older adults with normal nutritional status and at risk of malnutrition. The prevalence of low ASMI was 81.3% in older adults at risk of malnutrition and 20.6% for those with normal nutritional status (*P* < 0.0001).

**Table 1:** Characteristics of all participants.

	Overall (*n* = 1211)	Males (*n* = 504)	Females (*n* = 707)	*P V*alue (between genders)
Age (year)	73.20 (6.82)	73.39 (6.59)	73.06 (6.97)	0.4072
Ethnicity, *n* (%)				< 0.0001
Chinese	1036 (85.5)	404 (80.2)	632 (89.4)	
Non-Chinese	175 (14.5)	100 (19.8)	75 (10.6)	
Highest level of education, *n* (%)				0.0019
No formal education/primary	369 (30.6)	136 (27.0)	233 (33.1)	
Secondary O/N level or equivalent	507 (42.0)	201 (39.9)	306 (43.5)	
A level or equivalent	221 (18.3)	111 (22.0)	110 (15.6)	
University and above	110 (9.1)	56 (11.1)	54 (7.7)	
Smoking status, *n* (%)				< 0.0001
Non-smoker	946 (78.1)	277 (55.0)	669 (94.6)	
Past smoker	175 (14.5)	153 (30.4)	22 (3.1)	
Daily/occasional smoker	90 (7.4)	74 (14.7)	16 (2.3)	
Alcohol consumption, *n* (%)				< 0.0001
Non-drinker	729 (60.2)	237 (47.0)	492 (69.6)	
No drinks in last 12 months	210 (17.3)	117 (23.2)	93 (13.2)	
< Once a month in last 12 months	157 (13.0)	67 (13.3)	90 (12.7)	
≥ Once a month in last 12 months	115 (9.5)	83 (16.5)	32 (4.5)	
Hospital admission in last 6 months, *n* (%)				0.0010
Yes	112 (9.3)	63 (12.5)	49 (6.9)	
No	1098 (90.7)	441 (87.5)	657 (93.1)	
Days admitted in hospital last 6 months	0.74 (3.86)	1.06 (4.77)	0.52 (3.02)	0.0008
Number of prescribed drugs, *n* (%)				0.0340
Nil (0)	300 (24.8)	118 (23.4)	182 (25.7)	
One to five	697 (57.6)	280 (55.6)	417 (59.0)	
More than five (> 5)	214 (17.7)	106 (21.0)	108 (15.3)	
Physical Activity Scale for the Elderly score	109.05 (65.04)	112.77 (72.93)	106.40 (58.69)	0.1055
Modified Barthel Index score	98.71 (6.28)	98.92 (5.70)	98.56 (6.66)	0.3625
Modified Barthel Index, *n* (%)				0.7447
Severe dependence	9 (0.7)	3 (0.6)	6 (0.8)	
Moderate dependence	38 (3.1)	13 (2.6)	25 (3.5)	
Slight dependence	67 (5.5)	27 (5.4)	40 (5.7)	
Independent	1097 (90.6)	461 (91.5)	636 (90.0)	
Total Charlson Comorbidity score	0.06 (0.28)	0.10 (0.35)	0.04 (0.21)	0.0003
Charlson Comorbidity score, *n* (%)				0.0010
0	1142 (94.3)	461 (91.5)	681 (96.3)	
1	62 (5.1)	38 (7.5)	24 (3.4)	
2	5 (0.4)	3 (0.6)	2 (0.3)	
3	2 (0.2)	2 (0.4)	0	
25-hydroxyvitamin D (ug/L)	29.18 (9.69)	30.52 (10.13)	28.23 (9.26)	< 0.0001
25-hydroxyvitamin D, *n* (%)				0.0113
Deficient < 20 ug/L	203 (16.8)	67 (13.3)	136 (19.2)	
Insufficient 20– < 30 ug/L	487 (40.2)	201 (40.0)	286 (40.5)	
Sufficient 30–100 ug/L	520 (43.0)	235 (46.7)	285 (40.3)	
Height (cm)	157.30 (8.82)	164.44 (6.50)	152.21 (6.40)	< 0.0001
Body weight (kg)	50.78 (11.02)	55.67 (11.12)	47.29 (9.53)	< 0.0001
BMI (kg/m^2^)	20.43 (3.69)	20.52 (3.58)	20.37 (3.76)	0.4900
Mid upper arm circumference (cm)	24.44 (3.62)	25.13 (3.41)	23.95 (3.69)	< 0.0001
Calf circumference (cm)	32.07 (3.70)	33.08 (3.55)	31.35 (3.65)	< 0.0001
Bone mass (kg)	2.10 (0.45)	2.44 (0.34)	1.87 (0.36)	< 0.0001
Appendicular skeletal muscle mass (kg)	15.38 (4.03)	18.95 (3.54)	12.96 (2.07)	< 0.0001
Appendicular skeletal muscle mass index (kg/m^2^)	6.14 (1.13)	6.97 (1.09)	5.58 (0.75)	< 0.0001
Low appendicular skeletal muscle mass index, *n* (%)				0.1068
Yes	676 (59.9)	260 (57.0)	416 (61.8)	
No	453 (40.1)	196 (43.0)	257 (38.2)	

*BMI* body mass index, *O/N level* Ordinary/normal level, *A level* advanced level. For continuous variables, results are presented as mean (standard deviation). For categorical variables, results are presented as number (%).

### Characteristics by ASMI status

Table 2 shows the characteristics of the overall cohort, males, and females by their ASMI status. In the overall cohort, compared to those with normal ASMI, participants with low ASMI were older (74.30 years vs. 71.06 years), lighter (46.05 kg vs. 59.28 kg), with lower mid upper arm circumference (23.05 cm vs. 27.07 cm), calf circumference (30.47 cm vs. 34.94 cm), and bone mass (1.93 kg vs. 2.37 kg) (all *P* < 0.0001). There was a significant association between ASMI status and vitamin D status (*P* = 0.0472). Participants with low ASMI were likely to have vitamin D deficiency compared to those with normal ASMI (13.02% vs. 18.52%; *P* = 0.0144, data not shown). In addition, participants with low ASMI were almost twice as likely to have been admitted to the hospital within the prior 6 months (9.62% vs. 5.74%; *P* = 0.0190) and had significantly lower PASE scores (103.3 vs. 121.5; *P* < 0.0001). There was a significant difference in education level between the older adults with normal ASMI and low ASMI (*P* < 0.0001), with the latter having a higher percentage with no formal education (35.07% vs. 20.13%). Supplementary Table 2 shows the characteristics of older adults by their ASMI and nutritional status.

**Table 2:** Characteristics of all participants.

	Overall (*n* = 1129)			Males (*n* = 456)			Females (*n* = 673)		
	Normal ASMI (N = 453)	Low ASMI (N = 676)	*P v*alue	Normal ASMI (N = 196)	Low ASMI (N = 260)	*P v*alue	Normal ASMI (N = 257)	Low ASMI (N = 416)	*P v*alue
Appendicular skeletal muscle mass index (kg/m^2^)	7.02 (1.07)	5.55 (0.70)	< 0.0001	7.97 (0.78)	6.21 (0.54)	< 0.0001	6.30 (0.59)	5.13 (0.41)	< 0.0001
Appendicular skeletal muscle mass (kg)	17.82 (4.24)	13.75 (2.90)	< 0.0001	21.95 (2.75)	16.69 (2.08)	< 0.0001	14.67 (1.73)	11.91 (1.48)	< 0.0001
Age (year)	71.06 (5.13)	74.30 (7.25)	< 0.0001	71.24 (5.29)	74.72 (6.93)	< 0.0001	70.93 (5.01)	74.03 (7.43)	< 0.0001
Ethnicity, *n* (%)			0.8652			0.8891			0.8230
Chinese	385 (84.99)	577 (85.36)		155 (79.08)	207 (79.62)		230 (89.49)	370 (88.94)	
Non-Chinese	68 (15.01)	99 (14.64)		41 (20.92)	53 (20.38)		27 (10.51)	46 (11.06)	
Highest level of education, *n* (%)			< 0.0001			< 0.0001			0.0029
No formal education/primary	91 (20.13)	236 (35.07)		28 (14.29)	83 (31.92)		63 (24.61)	153 (37.05)	
Secondary O/N level or equivalent	198 (43.81)	278 (41.31)		81 (41.33)	102 (39.23)		117 (45.70)	176 (42.62)	
A level or equivalent	106 (23.45)	106 (15.75)		54 (27.55)	52 (20.00)		52 (20.31)	54 (13.08)	
University and above	57 (12.61)	53 (7.88)		33 (16.84)	23 (8.85)		24 (9.38)	30 (7.26)	
Smoking status, *n* (%)			0.0004			< 0.0001			0.1133
Non-smoker	375 (82.78)	521 (77.07)		129 (65.82)	129 (49.62)		246 (95.72)	392 (94.23)	
Past smoker	65 (14.35)	96 (14.20)		56 (28.57)	85 (32.69)		9 (3.50)	11 (2.64)	
Daily/Occasional smoker	13 (2.87)	59 (8.73)		11 (5.61)	46 (17.69)		2 (0.78)	13 (3.13)	
Alcohol consumption, *n* (%)			0.0263			0.0304			0.4032
Non-drinker	258 (68.80)	423 (74.87)		88 (57.89)	126 (62.07)		170 (76.23)	297 (82.04)	
No drinks in last 12 months	78 (17.22)	111 (16.42)		44 (22.45)	57 (21.92)		34 (13.23)	54 (12.98)	
< Once a month in last 12 months	76 (20.27)	73 (12.92)		37 (24.34)	26 (12.81)		39 (17.49)	47 (12.98)	
≥ Once a month in last 12 months	41 (10.93)	69 (12.21)		27 (17.76)	51 (25.12)		14 (6.28)	18 (4.97)	
Current marital status, *n* (%)			0.0001			0.0025			0.0511
Never married	49 (10.82)	110 (16.27)		13 (6.63)	34 (13.08)		36 (14.01)	76 (18.27)	
Currently married	325 (71.74)	402 (59.47)		167 (85.20)	186 (71.54)		158 (61.48)	216 (51.92)	
Separated/Divorced/Widowed	79 (17.44)	164 (24.26)		16 (8.16)	40 (15.38)		63 (24.51)	124 (29.81)	
Hospital admission in last 6 months, *n* (%)			0.0190			0.1534			0.0373
Yes	26 (5.74)	65 (9.62)		16 (8.16)	32 (12.31)		10 (3.89)	33 (7.93)	
No	427 (94.26)	611 (90.38)		180 (91.84)	228 (87.69)		247 (96.11)	383 (92.07)	
Days admitted in hospital last 6 months	0.37 (1.90)	0.78 (3.84)	0.0272	0.59 (2.35)	0.93 (4.04)	0.2315	0.21 (1.45)	0.68 (3.70)	0.0343
Number of prescribed drugs, *n* (%)			0.7464			0.0713			0.2551
Nil (0)	110 (24.28)	177 (26.18)		42 (21.43)	68 (26.15)		68 (26.46)	109 (26.20)	
One to five	262 (57.84)	385 (56.95)		105 (53.57)	149 (57.31)		157 (61.09)	236 (56.73)	
More than five (> 5)	81 (17.88)	114 (16.86)		49 (25.00)	43 (16.54)		32 (12.45)	71 (17.07)	
Physical Activity Scale for the Elderly score	121.52 (65.36)	103.25 (62.09)	< 0.0001	125.97 (73.24)	106.87 (69.15)	0.0046	118.13 (58.57)	100.99 (57.22)	0.0002
Modified Barthel Index score	99.37 (3.33)	98.63 (6.85)	0.2206	99.36 (2.97)	99.21 (5.32)	0.5176	99.37 (3.59)	98.27 (7.63)	0.0488
Total Charlson Comorbidity score	0.04 (0.24)	0.07 (0.29)	0.0724	0.06 (0.31)	0.10 (0.35)	0.1416	0.02 (0.15)	0.05 (0.23)	0.2219
25-hydroxyvitamin D (ug/L)	29.90 (9.80)	28.92 (9.63)	0.0989	32.01 (10.20)	30.12 (9.93)	0.0478	28.29 (9.17)	28.18 (9.38)	0.8837
25-hydroxyvitamin D (ug/L), *n* (%)			0.0472			0.0685			0.2472
Deficient < 20 ug/L	59 (13.02)	125 (18.52)		16 (8.16)	39 (15.06)		43 (16.73)	86 (20.67)	
Insufficient 20- < 30 ug/L	192 (42.38)	262 (38.81)		78 (39.80)	102 (39.38)		114 (44.36)	160 (38.46)	
Sufficient 30–100 ug/L	202 (44.59)	288 (42.67)		102 (52.04)	118 (45.56)		100 (38.91)	170 (40.87)	
Height (cm)	158.26 (8.77)	156.59 (8.75)	0.0017	165.76 (5.72)	163.70 (6.73)	0.0005	152.54 (5.92)	152.14 (6.68)	0.4185
Body weight (kg)	59.28 (11.06)	46.05 (6.78)	< 0.0001	65.36 (9.78)	49.91 (6.04)	< 0.0001	54.65 (9.65)	43.64 (6.08)	< 0.0001
BMI (kg/m^2^)	23.59 (3.54)	18.73 (1.97)	< 0.0001	23.75 (3.09)	18.60 (1.74)	< 0.0001	23.46 (3.85)	18.82 (2.10)	< 0.0001
Mid upper arm circumference (cm)	27.07 (3.58)	23.05 (2.46)	< 0.0001	27.72 (3.20)	23.66 (2.23)	< 0.0001	26.58 (3.78)	22.67 (2.52)	< 0.0001
Calf circumference (cm)	34.94 (3.22)	30.47 (2.69)	< 0.0001	35.95 (3.07)	31.42 (2.25)	< 0.0001	34.17 (3.12)	29.88 (2.78)	< 0.0001
Bone mass (kg)	2.37 (0.41)	1.93 (0.39)	< 0.0001	2.71 (0.27)	2.25 (0.24)	< 0.0001	2.11 (0.29)	1.73 (0.32)	< 0.0001

*BMI* body mass index, *O/N level* ordinary/normal level, *A level* advanced level. For continuous variables, results are presented as mean (standard deviation). For categorical variables, results are presented as number (%).

### Factors associated with ASMI

As shown in Table 3, in the overall cohort, factors associated with ASMI included age, gender, calf circumference, bone mass, and nutritional status. For every one-year increase in age while holding other factors constant, ASMI was significantly lower by − 0.011 (95% CI − 0.017, − 0.005). Females had significantly lower ASMI than males (− 0.792, 95% CI − 0.891, − 0.694). Compared to older adults with normal nutritional status, ASMI was significantly lower among those at medium risk (− 0.373, 95% CI − 0.460, − 0.286) and high risk (− 0.608, 95% CI − 0.713, − 0.503) of malnutrition. On the other hand, calf circumference and bone mass were positively associated with ASMI (both *P* < 0.0001). In addition, for both males and females, age and risk of malnutrition were negatively associated with ASMI, whereas calf circumference and bone mass were positively associated with ASMI (all *P* ≤ 0.0139).

**Table 3:** Factors associated with ASMI using multiple linear regression models: overall cohort, males and females.

	Overall Cohort (*n* = 1211)				Males (*n* = 504)				Females (*n* = 707)			
	Estimate (Beta)	Std. error	95% CI	*P v*alue	Estimate (Beta)	Std. error	95% CI	*P v*alue	Estimate (Beta)	Std. Error	95% CI	*P v*alue
Intercept	3.374	0.351	(2.685, 4.063)	< 0.0001	1.000	0.534	(− 0.050, 2.051)	0.0618	3.488	0.400	(2.702, 4.274)	< 0.0001
Age (year)	− 0.011	0.003	(− 0.017, − 0.005)	0.0002	− 0.011	0.004	(− 0.019, − 0.002)	0.0139	− 0.010	0.004	(− 0.016, − 0.003)	0.0059
Gender				< 0.0001								
Male (ref)	0				–	–	–	–	–	–	–	–
Female	− 0.792	0.050	(− 0.891, − 0.694)		–	–	–	–	–	–	–	–
Ethnicity				0.1591				0.3413				0.0237
Chinese (ref)	0				0				0			
Non-Chinese	0.067	0.047	(− 0.026, 0.160)		− 0.060	0.063	(− 0.183, 0.063)		0.147	0.065	(0.020, 0.274)	
Smoking				0.1595				0.6716				0.0566
Non-smoker (ref)	0				0				0			
Past smoker	0.039	0.053	(− 0.065, 0.143)		0.050	0.059	(− 0.065, 0.165)		0.243	0.112	(0.022, 0.463)	
Daily/Occasional smoker	− 0.112	0.071	(− 0.252, 0.028)		0.037	0.080	(− 0.120, 0.193)		− 0.130	0.134	(− 0.393, 0.133)	
Drinking				0.0509				0.0612				0.4182
Non-drinker (ref)	0				0				0			
No drinks in last 12 months	− 0.070	0.046	(− 0.160, 0.020)		− 0.0178	0.0655	(− 0.147, 0.111)		− 0.055	0.058	(− 0.168, 0.059)	
< Once a month in last 12 months	− 0.106	0.051	(− 0.205, − 0.007)		− 0.188	0.077	(− 0.340, − 0.036)		− 0.077	0.059	(− 0.193, 0.039)	
≥ Once a month in last 12 months	− 0.123	0.059	(− 0.238, − 0.008)		− 0.1145	0.0717	(− 0.255, 0.027)		− 0.092	0.092	(− 0.272, 0.088)	
Marital status				0.8135				0.6322				0.8732
Currently married (ref)	0				0				0			
Separated/Divorced/Widowed	0.026	0.043	(− 0.059, 0.111)		0.074	0.077	(− 0.078, 0.226)		− 0.014	0.048	(− 0.107, 0.080)	
Never married	− 0.004	0.048	(− 0.099, 0.090)		0.007	0.082	(− 0.154, 0.167)		0.019	0.054	(− 0.087, 0.124)	
Education				0.1081				0.0606				0.0690
No formal education/primary (ref)	0				0				0			
Secondary O/N level or equivalent	− 0.064	0.041	(− 0.145, 0.017)		0.093	0.066	(− 0.036, 0.222)		− 0.099	0.048	(− 0.193, − 0.005)	
A level or equivalent	− 0.098	0.050	(− 0.197, 0.000)		− 0.050	0.074	(− 0.196, 0.095)		− 0.110	0.061	(− 0.231, 0.010)	
University and above	− 0.135	0.063	(− 0.259, − 0.011)		− 0.075	0.090	(− 0.252, 0.102)		− 0.180	0.079	(− 0.335, − 0.025)	
Physical Activity Scale for the Elderly score	0.0003	0.0003	(− 0.0002, 0.0009)	0.2184	0.0003	0.0004	(− 0.0004, 0.0010)	0.4675	0.0004	0.0004	(− 0.0003, 0.0011)	0.2699
Calf circumference (cm)	0.085	0.007	(0.071, 0.099)	< 0.0001	0.123	0.011	(0.101, 0.145)	< 0.0001	0.066	0.008	(0.050, 0.082)	< 0.0001
Bone mass (kg)	0.778	0.067	(0.646, 0.909)	< 0.0001	1.193	0.112	(0.972, 1.414)	< 0.0001	0.542	0.076	(0.393, 0.690)	< 0.0001
MUST risk category				< 0.0001				< 0.0001				< 0.0001
Low (ref)	0				0				0			
Medium	− 0.373	0.044	(− 0.460, − 0.286)		− 0.388	0.068	(− 0.521, − 0.255)		− 0.298	0.052	(− 0.401, − 0.195)	
High	− 0.608	0.054	(− 0.713, − 0.503)		− 0.579	0.084	(− 0.744, − 0.415)		− 0.554	0.062	(− 0.676, − 0.432)	

*O/N level* ordinary/normal level, *A level* advanced level, *MUST* Malnutrition universal screening test.

### Factors associated with low ASMI

Multiple logistic regression model showed that factors associated with low ASMI included age, smoking, drinking, calf circumference, bone mass, and nutritional status (Table 4). With every one-year increase in age, the odds of having low ASMI increased by 6% (odds ratio: 1.06, 95% CI 1.02, 1.09). Compared to non-smoker, daily or occasional smokers had significantly higher odds of having low ASMI (odds ratio: 3.76, 95% CI 1.55, 9.16). The odds of having low ASMI for older adults who drank at least once a month was 2.51 (95% CI 1.34, 4.67), compared to non-drinkers. Calf circumference and bone mass were associated with lower odds of having low ASMI (both *P* < 0.0001). Compared to older adults with normal nutritional status, the odds ratio of having low ASMI was 3.58 (95% CI 2.41, 5.30) among those at medium risk of malnutrition, and 12.50 (95% CI 7.02, 22.25) among those at high risk of malnutrition. In addition, for both males and females, calf circumference and bone mass were associated with lower odds of having low ASMI (all *P* < 0.0001). On the other hand, malnutrition risk was associated with higher odds of having low ASMI in both genders (both *P* < 0.0001), and age for females only (*P* = 0.0425).

**Table 4:** Factors associated with low ASMI using multiple logistic regression models: overall cohort, males and females.

	Overall Cohort (*n* = 1211)			Males (*n* = 504)			Females (*n* = 707)		
	Odds ratio	95% CI	*P v*alue	Odds ratio	95% CI	*P v*alue	Odds ratio	95% CI	*P v*alue
Age (year)	1.06	(1.02, 1.09)	0.0011	1.00	(0.94, 1.06)	0.9019	1.05	(1.00, 1.09)	0.0425
Ethnicity			0.3151			0.5679			0.7929
Chinese (ref)	1.00			1.00			1.00		
Non-Chinese	1.31	(0.77, 2.22)		1.30	(0.53, 3.21)		0.90	(0.40, 2.01)	
Smoking			0.0134			0.4439			0.8041
Non-smoker (ref)	1.00			1.00			1.00		
Past smoker	1.18	(0.69, 2.03)		0.74	(0.34, 1.61)		0.83	(0.22, 3.10)	
Daily/Occasional smoker	3.76	(1.55, 9.16)		1.81	(0.51, 6.42)		1.94	(0.22, 16.97)	
Drinking			0.0328			0.2856			0.3608
Non-drinker (ref)	1.00			1.00			1.00		
No drinks in last 12 months	1.40	(0.86, 2.26)		0.88	(0.38, 2.04)		1.64	(0.85, 3.15)	
< Once a month in last 12 months	1.28	(0.74, 2.21)		1.29	(0.44, 3.81)		1.49	(0.76, 2.95)	
≥ Once a month in last 12 months	2.51	(1.34, 4.67)		2.35	(0.87, 6.36)		1.48	(0.54, 4.04)	
Marital status			0.4346			0.9584			0.9713
Currently married (ref)	1.00			1.00			1.00		
Separated/Divorced/Widowed	0.74	(0.46, 1.17)		1.10	(0.35, 3.47)		0.94	(0.54, 1.62)	
Never married	0.92	(0.55, 1.56)		0.87	(0.27, 2.84)		0.95	(0.51, 1.80)	
Education			0.2757			0.2228			0.0914
No formal education/primary (ref)	1.00			1.00			1.00		
Secondary O/N level or equivalent	1.22	(0.78, 1.90)		0.84	(0.35, 2.04)		1.34	(0.77, 2.32)	
A level or equivalent	1.00	(0.59, 1.71)		2.03	(0.73, 5.61)		0.71	(0.36, 1.40)	
University and above	1.80	(0.94, 3.47)		1.74	(0.56, 5.35)		2.03	(0.83, 4.97)	
Physical Activity Scale for the Elderly score	0.999	(0.996, 1.001)	0.3094	0.998	(0.993, 1.003)	0.3716	0.998	(0.993, 1.002)	0.2311
Calf circumference (cm)	0.72	(0.66, 0.78)	< 0.0001	0.69	(0.59, 0.82)	< 0.0001	0.73	(0.66, 0.81)	< 0.0001
Bone mass (kg)	0.26	(0.15, 0.47)	< 0.0001	0.01	(0.00, 0.04)	< 0.0001	0.12	(0.05, 0.30)	< 0.0001
MUST risk category			< 0.0001			< 0.0001			< 0.0001
Low (ref)	1.00			1.00			1.00		
Medium	3.58	(2.41, 5.31)		5.69	(2.71, 11.96)		2.24	(1.36, 3.72)	
High	12.50	(7.02, 22.25)		14.18	(4.73, 42.56)		8.54	(4.16, 17.55)	

*O/N level* ordinary/normal level, *A level* advanced level, *MUST* malnutrition universal screening test.

### Calf circumference cut-offs

**Figure 2:**
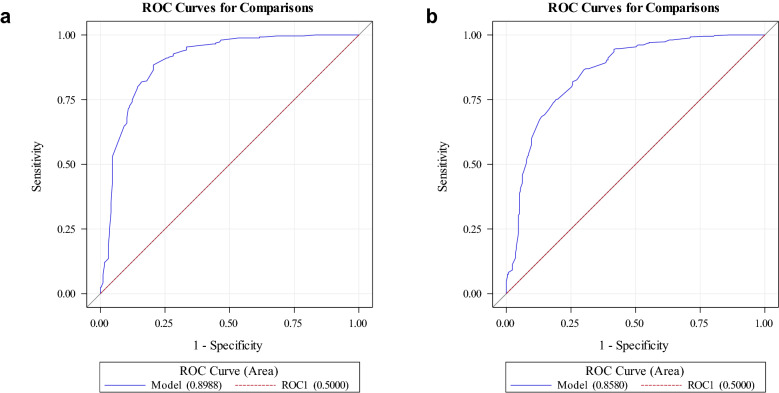
Receiver operating characteristic curves of calf circumference for low muscle mass in (**a**) males; and (**b**) females.

The cut-off values of calf circumference for low ASMI for males was 33.4 cm (sensitivity = 82%, specificity = 82%) and for females was 32.2 cm (sensitivity = 82%, specificity = 74%). As shown in Fig. 2, the area under the ROC curve for males was 0.8988, and for females was 0.8580. The Kappa statistics showed strong agreement between the cut-off values of calf circumference for low ASMI derived from this study and the cut-off values for screening for sarcopenia as recommended by the AWGS (males = 0.88, females = 0.87; both *P* < 0.0001).

## Discussion

To our knowledge, this is a first-of-kind study that determined the prevalence of low ASMI and its associated factors in community-dwelling, ambulant older adults. The findings of our study revealed that low ASMI was especially common among this cohort. We further identified factors that were associated with increased risk for low ASMI, i.e., age, malnutrition or its risk, smoking, alcohol consumption, low calf circumference, and low bone mass. These findings can be used to promote public health programs that can help older adults restore and maintain physical function, thus sustaining independent living in the community.

### Gender

Men are known to have more muscle mass than women, both in terms of absolute amount as well as in relation to overall composition^9^. This is predominantly due to the difference in muscle mass in the upper body. However, both genders experienced the same decline in muscle mass with aging, which is noticeable after the fifth decade^9^. The literature has also reported gender-specific differences in mid-upper arm circumference^38^, calf circumference^39^, and ASMI^1,2^. These differences between genders are also reflected in the present study.

### Older age

Older age was a key factor in predicting low ASMI. Our findings showed that there was a 6% increase in the odds of having low ASMI for every year older above 65. While age is a non-modifiable risk factor, it serves as an important marker for low ASMI.

This association between age and low ASMI is also reflected in the association between age and sarcopenia^40,41^. This is because low muscle mass is one of the diagnostic criteria for sarcopenia. In fact, researchers associated the highest likelihood of sarcopenia in the oldest old^5,42^, with older adults aged ≥ 70 years reported to lose up to 15% of muscle mass every decade^43^. In addition, participants aged ≥ 80 years were six times more likely to be at risk of sarcopenia as compared to those aged 60–80 years^40^. This finding is reflected in results from a Singapore study, which showed that more than one in two older adults above 80 years old will be at risk of losing at least one instrumental activity of daily living and adversely affect the ability to live independently^44^.

### Risk of malnutrition

In our study, low ASMI was significantly and prominently correlated with poor nutritional status. The odds of having low ASMI for older adults at high risk of malnutrition was 12.5 compared to their nourished counterparts, after adjusting for potential confounders. In fact, low muscle mass is now considered a defining characteristic of malnutrition^45^. The association between low muscle mass and malnutrition has been reported in the literature^46–48^. According to Landi et al., malnutrition needs to be addressed clinically as a muscle-related disorder, and clinicians should integrate nutrition assessment with muscle mass measurements for optimal evaluation of these two interrelated entities^48^. In parallel, Deutz et al. advised clinicians to place muscle mass at the core of nutrition assessment and management strategies^47^.

Our findings of low muscle mass are also in line with a recent study, which reported that older adults with malnutrition or at risk of malnutrition were 13.6 times more likely to be at risk of sarcopenia, compared to those with normal nutritional status^40^. In another study of community-dwelling older adults in Singapore, risk of malnutrition was strongly associated with sarcopenia (odds ratio = 9.9)^49^. Similarly, a cohort study found that malnutrition was associated with an approximately four-fold higher risk of sarcopenia developing during a four-year follow-up^50^. Community-dwelling older Chinese adults with malnutrition experienced adverse outcomes such as sarcopenia, frailty, and mortality at 14-year follow-up^51^.

These observations suggest that assessing the risk for malnutrition in community-dwelling older adults could be a useful strategy for early detection of individuals at risk of low ASMI. Validated tools such as MUST and Mini Nutritional Assessment - Short Form can be used to assess the risk of malnutrition^52,53^.

### Bone mass

Our findings of a direct correlation between low ASMI and low bone mass in older people are also in line with other studies in this area^54,55^. Specifically, in a Chinese population, leg lean mass was the strongest factor for prediction of femur bone mass^54^. Reduction in muscle strength over time was found to be the most significant independent factor associated with bone loss for Korean men and women^56^. Taken together, all these findings are in line with the established link between skeletal muscle and bone health.

### Lifestyle-related risk factors

In the present study, we found that people with low ASMI had lower physical activity scores than those with normal ASMI. In addition, smoking and drinking were associated with low ASMI. Previous research reported that lifestyle-related risks for low muscle mass include physical inactivity^20^, smoking^21^, and drinking^14^. Low physical activity leads to muscle decline due to disuse atrophy^57,58^. Smoking may impair muscle protein synthesis, while chronic alcoholic myopathy can occur in up to 50% of alcohol misusers leading to reduction of muscle mass and strength^59^. All three of these factors are potentially modifiable, so risk can be lessened by increasing physical activity, smoking cessation, and reducing alcohol consumption.

### Calf circumference cut-off values for low ASMI

Previous research showed that low muscle mass was significantly associated with low calf circumference in older people^60–62^. In the present study, we used ROC curve analysis to determine calf circumference cut-off values for low ASMI, i.e., 33.4 cm for males and 32.2 cm for females. A previous study found that calf circumference was positively correlated with appendicular skeletal muscle mass and skeletal muscle index, with cut-off values of calf circumference for predicting low muscle mass as < 34 cm in men and < 33 cm in women in a large study in Japan (*n* = 526)^60^. Similarly, a large study of older adults in Korea (*n* = 657 adults aged 70–84 years) found optimal cut-off values of calf circumference for low muscle mass as 35 cm for males and 33 cm for females^61^. Importantly, low calf circumference based on these cut-off values was associated with poor physical function^61^. Overall, these findings are in line with the calf circumference cut-offs for screening for sarcopenia from AWGS (< 34 cm in men and < 33 cm in women)^1^, thus supporting calf measurement as a screening tool for both low ASMI and sarcopenia in the community.

In the community, initial screening for low muscle mass can be done by measuring calf circumference^60–62^. We have established the cut-off values for low ASMI in community-dwelling older adults in Singapore, with strong agreement with the AWGS cut-off values for screening for sarcopenia (Kappa statistics for males = 0.88, for females = 0.87). As the measurement of calf circumference is low cost, easy to perform, and non-invasive, it can potentially be an ideal screening tool both in the community and clinical settings.

### Strengths and limitations

Our study has multiple strengths in design and outcomes, including (1) a large population of 1211 community-dwelling older adults, (2) extensive data with a full picture of interacting factors, (3) observation of significant associations between risk of malnutrition and low ASMI after adjusting for potential confounders, and (4) establishment of gender-specific calf circumference cut-off values for low ASMI. As for limitations, this is a cross-sectional study, so it shows associations between low ASMI and certain health and demographic factors, but it does not prove causal relationship. Since our population size was substantial, and these associations were statistically significant, we propose that healthcare professionals use these findings to identify aging individuals at risk of low ASMI. In addition, the results of this study in the multiethnic Singapore population may be applicable to other Asian populations.

## Conclusions

In conclusion, results of our study showed a high prevalence of low ASMI in community-dwelling older adults in Singapore, particularly among those at risk of malnutrition. The odds of having low ASMI was 12.5 for older adults at high risk of malnutrition compared to their nourished counterparts. For community-dwelling older adults, early identification of low muscle mass offers opportunities to prevent or delay its worsening, and its associated adverse consequences. We advise identification of malnutrition risk as a targeted core strategy to screen for low muscle mass in older adults.

Other risk factors included smaller calf circumference, older age, and lower bone mass, which were also significantly associated with risk of low ASMI. We have also determined gender-specific cut-off values of calf circumference for low ASMI in this population group and may thus serve as an easy-to-measure screening tool for low ASMI. Furthermore, low ASMI was associated with modifiable risk factors such as smoking and drinking, which suggests that current targeted community health promotion programs can improve muscle health, in addition to other established benefits.

Early identification of risk for low ASMI in older adults can guide appropriate interventions, which can in turn reduce associated health complications, lower healthcare costs, improve function and quality of life, and thus supporting healthier, more independent and meaningful lives for as long as possible.

## Supplementary Information

Supplementary Information.

## Data Availability

The data presented in this study are available within the paper and its Supplementary Dataset.
