# Supplementary material for: Prevalence of low muscle mass and associated factors in community-dwelling older adults in Singapore
Source: Sci Rep. 2021 Nov 29;11:23071. doi: 10.1038/s41598-021-02274-3 (PMC8630119; doi:10.1038/s41598-021-02274-3)
Supplement: Supplementary file 1 — Supplementary Information. [file 41598_2021_2274_MOESM1_ESM.docx]

Prevalence of low muscle mass and associated factors in community-dwelling older adults in Singapore

Siew Ling Tey^1,*^, Dieu Thi Thu Huynh^1^, Yatin Berde^2^, Geraldine Baggs^3^, Choon How How^4,5^, Yen Ling Low^1^, Magdalin Cheong^6^, Wai Leng Chow^7^, Ngiap Chuan Tan^5,8^ and Samuel Teong Huang Chew^9,10^

^1^Abbott Nutrition Research and Development, Asia-Pacific Center, Singapore 138668, Singapore. ^2^Statistical Services, Cognizant Technologies Solution Pvt. Ltd. Mumbai, India. ^3^Abbott Nutrition Research and Development, Columbus, OH 43219, USA. ^4^Care and Health Integration, Changi General Hospital, Singapore 529889, Singapore. ^5^SingHealth-Duke NUS Family Medicine Academic Clinical Program, Duke-NUS Medical School, Singapore 169857, Singapore. ^6^Department of Dietetic & Food Services, Changi General Hospital, Singapore 529889, Singapore. ^7^Health Services Research, Changi General Hospital, Singapore. ^8^SingHealth Polyclinics, Singapore 150167, Singapore. ^9^Department of Geriatric Medicine, Changi General Hospital, Singapore 529889, Singapore. ^10^Yong Loo Lin School of Medicine, National University of Singapore, Singapore 117597, Singapore. email: siewling.tey@abbott.com.

**Supplementary Table 1.** Characteristics of all participants by nutritional status

|  | Overall  (*n* = 1211) | Normal nutrition  (*n* = 400) | Risk of malnutrition  (*n* = 811) | *p v*alue  (between nutritional status) |
| --- | --- | --- | --- | --- |
| Age (year) | 73.20 (6.82) | 71.21 (5.27) | 74.18 (7.27) | <0.0001 |
| Ethnicity, *n* (%) |  |  |  | 0.0764 |
| Chinese | 1036 (85.5) | 332 (83.0) | 704 (86.8) |  |
| Non-Chinese | 175 (14.5) | 68 (17.0) | 107 (13.2) |  |
| Highest level of education, *n* (%) |  |  |  | <0.0001 |
| No formal education / primary | 369 (30.6) | 62 (15.5) | 307 (38.0) |  |
| Secondary O/N level or equivalent | 507 (42.0) | 186 (46.5) | 321 (39.8) |  |
| A level or equivalent | 221 (18.3) | 100 (25.0) | 121 (15.0) |  |
| University and above | 110 (9.1) | 52 (13.0) | 58 (7.2) |  |
| Smoking status, *n* (%) |  |  |  | <0.0001 |
| Non-smoker | 946 (78.1) | 333 (83.3) | 613 (75.6) |  |
| Past smoker | 175 (14.5) | 57 (14.3) | 118 (14.5) |  |
| Daily/Occasional smoker | 90 (7.4) | 10 (2.5) | 80 (9.9) |  |
| Alcohol consumption, *n* (%) |  |  |  | 0.0022 |
| Non-drinker | 729 (60.2) | 214 (53.5) | 515 (63.5) |  |
| No drinks in last 12 months | 210 (17.3) | 73 (18.3) | 137 (16.9) |  |
| <Once a month in last 12 months | 157 (13.0) | 69 (17.3) | 88 (10.9) |  |
| ≥Once a month in last 12 months | 115 (9.5) | 44 (11.0) | 71 (8.8) |  |
| Hospital admission in last 6 months, *n* (%)  Yes  No | 112 (9.3)  1098 (90.7) | 18 (4.5)  382 (95.5) | 94 (11.6)  716 (88.4) | <0.0001 |
| Days admitted in hospital last 6 months | 0.74 (3.86) | 0.34 (2.04) | 0.94 (4.48) | <0.0001 |
| Number of prescribed drugs, *n* (%) |  |  |  |  |
| Nil (0) | 300 (24.8) | 86 (21.5) | 214 (26.4) | 0.1554 |
| One to five | 697 (57.6) | 237 (59.3) | 460 (56.7) |  |
| More than five (>5) | 214 (17.7) | 77 (19.3) | 137 (16.9) |  |
| Physical Activity Scale for the Elderly score | 109.05 (65.04) | 119.45 (63.44) | 103.92 (65.25) | <0.0001 |
| Modified Barthel Index score | 98.71 (6.28) | 99.54 (2.39) | 98.30 (7.45) | 0.0303 |
| Modified Barthel Index, *n* (%) |  |  |  | 0.0355 |
| Severe dependence | 9 (0.7) | 0 | 9 (1.1) |  |
| Moderate dependence | 38 (3.1) | 7 (1.8) | 31 (3.8) |  |
| Slight dependence | 67 (5.5) | 21 (5.3) | 46 (5.7) |  |
| Independent | 1097 (90.6) | 372 (93.0) | 725 (89.4) |  |
| Total Charlson Comorbidity score | 0.06 (0.28) | 0.03 (0.22) | 0.08 (0.31) | 0.0001 |
| Charlson Comorbidity score, *n* (%) |  |  |  | <0.0001 |
| 0 | 1142 (94.3) | 392 (98.0) | 750 (92.5) |  |
| 1 | 62 (5.1) | 6 (1.5) | 56 (6.9) |  |
| 2 | 5 (0.4) | 1 (0.3) | 4 (0.5) |  |
| 3 | 2 (0.2) | 1 (0.3) | 1 (0.1) |  |
| 25-hydroxyvitamin D (ug/L) | 29.18 (9.69) | 30.42 (10.14) | 28.57 (9.41) | 0.0018 |
| 25-hydroxyvitamin D, *n* (%) |  |  |  | 0.0204 |
| Deficient <20 ug/L | 203 (16.8) | 54 (13.5) | 149 (18.4) |  |
| Insufficient 20 - <30 ug/L | 487 (40.2) | 154 (38.5) | 333 (41.1) |  |
| Sufficient 30 - 100 ug/L | 520 (43.0) | 192 (48.0) | 328 (40.5) |  |
| Height (cm) | 157.30 (8.82) | 158.68 (8.51) | 156.62 (8.90) | 0.3023 |
| Body weight (kg) | 50.78 (11.02) | 61.86 (9.67) | 45.31 (6.69) | 0.0001 |
| BMI (kg/m^2^) | 20.43 (3.69) | 24.53 (3.08) | 18.41 (1.80) | <0.0001 |
| Mid upper arm circumference (cm) | 24.44 (3.62) | 27.73 (3.27) | 22.81 (2.50) | <0.0001 |
| Calf circumference (cm) | 32.07 (3.70) | 35.24 (3.21) | 30.50 (2.82) | <0.0001 |
| Bone mass (kg) | 2.10 (0.45) | 2.38 (0.42) | 1.96 (0.40) | <0.0001 |
| Appendicular skeletal muscle mass (kg) | 15.38 (4.03) | 17.75 (4.41) | 14.09 (3.13) | <0.0001 |
| Appendicular skeletal muscle mass index (kg/m^2^) | 6.14 (1.13) | 6.96 (1.19) | 5.69 (0.80) | <0.0001 |
| Low appendicular skeletal muscle mass index, *n* (%) |  |  |  | <0.0001 |
| Yes | 676 (59.9) | 82 (20.6) | 594 (81.3) |  |
| No | 453 (40.1) | 316 (79.4) | 137 (18.7) |  |

O/N level, General Certificate of Education: Ordinary Level / Normal Level; A level, General Certificate of Education: Advanced Level; BMI, body mass index. For continuous variables, results are presented as mean (standard deviation). For categorical variables, results are presented as number (%).

**Supplementary Table 2.** Characteristics of all participants by ASMI and nutritional status

|  | Overall (*n* = 1129) | | | Normal nutrition (*n* = 398) | | | Risk of malnutrition (*n* = 731) | | |
| --- | --- | --- | --- | --- | --- | --- | --- | --- | --- |
|  | Normal ASMI  (*n* = 453) | Low ASMI  (*n* = 676) | *p* value | Normal ASMI  (*n* = 316) | Low ASMI  (*n* = 82) | *p* value | Normal ASMI  (*n* = 137) | Low ASMI  (*n* = 594) | *p* value |
| Appendicular skeletal muscle mass index (kg/m^2^) | 7.02 (1.07) | 5.55 (0.70) | <0.0001 | 7.27 (1.10) | 5.77 (0.62) | <0.0001 | 6.45 (0.74) | 5.52 (0.71) | <0.0001 |
| Appendicular skeletal muscle mass (kg) | 17.82 (4.24) | 13.75 (2.90) | <0.0001 | 18.65 (4.30) | 14.28 (2.88) | <0.0001 | 15.90 (3.43) | 13.67 (2.90) | <0.0001 |
| Age (year) | 71.06 (5.13) | 74.30 (7.25) | <0.0001 | 70.61 (4.83) | 73.56 (6.25) | 0.0001 | 72.10 (5.65) | 74.40 (7.37) | <0.0001 |
|  |  |  |  |  |  |  |  |  |  |
| Ethnicity, *n* (%) |  |  | 0.8652 |  |  | 0.7444 |  |  | 0.3249 |
| Chinese | 385 (84.99) | 577 (85.36) |  | 263 (83.23) | 67 (81.71) |  | 122 (89.05) | 510 (85.86) |  |
| Non-Chinese | 68 (15.01) | 99 (14.64) |  | 53 (16.77) | 15 (18.29) |  | 15 (10.95) | 84 (14.14) |  |
| Highest level of education, *n* (%) |  |  | <0.0001 |  |  | 0.4972 |  |  | 0.0093 |
| No formal education / primary | 91 (20.13) | 236 (35.07) |  | 48 (15.19) | 14 (17.07) |  | 43 (31.62) | 222 (37.56) |  |
| Secondary O/N level or equivalent | 198 (43.81) | 278 (41.31) |  | 152 (48.10) | 33 (40.24) |  | 46 (33.82) | 245 (41.46) |  |
| A level or equivalent | 106 (23.45) | 106 (15.75) |  | 74 (23.42) | 25 (30.49) |  | 32 (23.53) | 81 (13.71) |  |
| University and above | 57 (12.61) | 53 (7.88) |  | 42 (13.29) | 10 (12.20) |  | 15 (11.03) | 43 (7.28) |  |
| Smoking status, *n* (%) |  |  | 0.0004 |  |  | 0.6819 |  |  | 0.0338 |
| Non-smoker | 375 (82.78) | 521 (77.07) |  | 262 (82.91) | 70 (85.37) |  | 113 (82.48) | 451 (75.93) |  |
| Past smoker | 65 (14.35) | 96 (14.20) |  | 45 (14.24) | 11 (13.41) |  | 20 (14.60) | 85 (14.31) |  |
| Daily/Occasional smoker | 13 (2.87) | 59 (8.73) |  | 9 (2.85) | 1 (1.22) |  | 4 (2.92) | 58 (9.76) |  |
| Alcohol consumption, *n* (%) |  |  | 0.0263 |  |  | 0.1583 |  |  | 0.9551 |
| Non-drinker | 258 (68.80) | 423 (74.87) |  | 170 (65.38) | 43 (66.15) |  | 88 (76.52) | 380 (76.00) |  |
| No drinks in last 12 months | 78 (17.22) | 111 (16.42) |  | 56 (17.72) | 17 (20.73) |  | 22 (16.06) | 94 (15.82) |  |
| <Once a month in last 12 months | 76 (20.27) | 73 (12.92) |  | 60 (23.08) | 9 (13.85) |  | 16 (13.91) | 64 (12.80) |  |
| ≥Once a month in last 12 months | 41 (10.93) | 69 (12.21) |  | 30 (11.54) | 13 (20.00) |  | 11 (9.57) | 56 (11.20) |  |
| Current marital status, *n* (%) |  |  | 0.0001 |  |  | 0.3352 |  |  | 0.1772 |
| Never married | 49 (10.82) | 110 (16.27) |  | 26 (8.23) | 11 (13.41) |  | 23 (16.79) | 99 (16.67) |  |
| Currently married | 325 (71.74) | 402 (59.47) |  | 236 (74.68) | 59 (71.95) |  | 89 (64.96) | 343 (57.74) |  |
| Separated/Divorced/Widowed | 79 (17.44) | 164 (24.26) |  | 54 (17.09) | 12 (14.63) |  | 25 (18.25) | 152 (25.59) |  |
| Hospital admission in last 6 months, *n* (%) |  |  | 0.0190 |  |  | 0.3571 |  |  | 0.3674 |
| Yes | 26 (5.74) | 65 (9.62) |  | 15 (4.75) | 2 (2.44) |  | 11 (8.03) | 63 (10.61) |  |
| No | 427 (94.26) | 611 (90.38) |  | 301 (95.25) | 80 (97.56) |  | 126 (91.97) | 531 (89.39) |  |
| Days admitted in hospital last 6 months | 0.37 (1.90) | 0.78 (3.84) | 0.0272 | 0.34 (1.99) | 0.30 (2.27) | 0.3700 | 0.44 (1.68) | 0.84 (4.00) | 0.5006 |
| Number of prescribed drugs, *n* (%) |  |  | 0.7464 |  |  | 0.7712 |  |  | 0.6388 |
| Nil (0) | 110 (24.28) | 177 (26.18) |  | 68 (21.52) | 18 (21.95) |  | 42 (30.66) | 159 (26.77) |  |
| One to five | 262 (57.84) | 385 (56.95) |  | 189 (59.81) | 46 (56.10) |  | 73 (53.28) | 339 (57.07) |  |
| More than five (>5) | 81 (17.88) | 114 (16.86) |  | 59 (18.67) | 18 (21.95) |  | 22 (16.06) | 96 (16.16) |  |
| Physical Activity Scale for the Elderly score | 121.52 (65.36) | 103.25 (62.09) | <0.0001 | 122.08 (65.70) | 108.91 (53.53) | 0.0610 | 120.25 (64.80) | 102.47 (63.19) | 0.0032 |
| Modified Barthel Index score | 99.37 (3.33) | 98.63 (6.85) | 0.2206 | 99.48 (2.61) | 99.74 (1.27) | 0.6875 | 99.09 (4.58) | 98.48 (7.28) | 0.4783 |
| Total Charlson Comorbidity score | 0.04 (0.24) | 0.07 (0.29) | 0.0724 | 0.03 (0.21) | 0.02 (0.22) | 0.6858 | 0.07 (0.29) | 0.07 (0.29) | 0.8829 |
| 25-hydroxyvitamin D (ug/L) | 29.90 (9.80) | 28.92 (9.63) | 0.0989 | 30.62 (10.07) | 29.82 (10.49) | 0.5286 | 28.24 (8.93) | 28.80 (9.51) | 0.5290 |
| 25-hydroxyvitamin D, *n* (%) |  |  | 0.0472 |  |  | 0.3693 |  |  | 0.0591 |
| Deficient <20 ug/L | 59 (13.02) | 125 (18.52) |  | 39 (12.34) | 15 (18.29) |  | 20 (14.60) | 110 (18.55) |  |
| Insufficient 20 - <30 ug/L | 192 (42.38) | 262 (38.81) |  | 123 (38.92) | 29 (35.37) |  | 69 (50.36) | 233 (39.29) |  |
| Sufficient 30 - 100 ug/L | 202 (44.59) | 288 (42.67) |  | 154 (48.73) | 38 (46.34) |  | 48 (35.04) | 250 (42.16) |  |
| Height (cm) | 158.26 (8.77) | 156.59 (8.75) | 0.0017 | 159.21 (8.48) | 156.55 (8.44) | 0.0116 | 156.07 (9.08) | 156.59 (8.80) | 0.5312 |
| Body weight (kg) | 59.28 (11.06) | 46.05 (6.78) | <0.0001 | 63.84 (9.31) | 53.87 (6.09) | <0.0001 | 48.76 (6.77) | 44.97 (6.14) | <0.0001 |
| BMI (kg/m^2^) | 23.59 (3.54) | 18.73 (1.97) | <0.0001 | 25.16 (3.00) | 21.97 (1.71) | <0.0001 | 19.95 (1.35) | 18.29 (1.55) | <0.0001 |
| Mid upper arm circumference (cm) | 27.07 (3.58) | 23.05 (2.46) | <0.0001 | 28.33 (3.23) | 25.40 (2.33) | <0.0001 | 24.14 (2.49) | 22.72 (2.29) | <0.0001 |
| Calf circumference (cm) | 34.94 (3.22) | 30.47 (2.69) | <0.0001 | 35.94 (2.99) | 32.45 (2.36) | <0.0001 | 32.62 (2.46) | 30.20 (2.62) | <0.0001 |
| Bone mass (kg) | 2.37 (0.41) | 1.93 (0.39) | <0.0001 | 2.47 (0.40) | 2.06 (0.36) | <0.0001 | 2.15 (0.36) | 1.91 (0.39) | <0.0001 |

O/N level, General Certificate of Education: Ordinary Level / Normal Level; A level, General Certificate of Education: Advanced Level; BMI, body mass index. For continuous variables, results are presented as mean (standard deviation). For categorical variables, results are presented as number (%).
